# Bioaerosols Play a Major Role in the Nasopharyngeal Microbiota Content in Agricultural Environment

**DOI:** 10.3390/ijerph16081375

**Published:** 2019-04-16

**Authors:** Hamza Mbareche, Marc Veillette, Jonathan Pilote, Valérie Létourneau, Caroline Duchaine

**Affiliations:** 1 Centre de Recherche de l’Institut Universitaire de Cardiologie et de Pneumologie de Québec, Québec, QC G1V 4G5, Canada; hamza.mbareche.1@ulaval.ca (H.M.); Marc.Veillette@criucpq.ulaval.ca (M.V.); Jonathan.pilote.31@gmail.com (J.P.); Valerie.Letourneau@criucpq.ulaval.ca (V.L.); 2 Département de Biochimie, de Microbiologie et de Bio-Informatique, Faculté des Sciences et de Génie, Université Laval, Québec, QC G1V 0A6, Canada

**Keywords:** bioaerosols, air quality, nasopharynx, occupational exposure, microbial diversity, resistance genes, high-throughput sequencing

## Abstract

*Background*: Bioaerosols are a major concern for public health and sampling for exposure assessment purposes is challenging. The nasopharyngeal region could be a potent carrier of long-term bioaerosol exposure agents. This study aimed to evaluate the correlation between nasopharyngeal bacterial flora of swine workers and the swine barns bioaerosol biodiversity. *Methods*: Air samples from eight swine barns as well as nasopharyngeal swabs from pig workers (*n* = 25) and from a non-exposed control group (*n* = 29) were sequenced using 16S rRNA gene high-throughput sequencing. Wastewater treatment plants were used as the industrial, low-dust, non-agricultural environment control to validate the microbial link between the bioaerosol content (air) and the nasopharynxes of workers. *Results*: A multivariate analysis showed air samples and nasopharyngeal flora of pig workers cluster together, compared to the non-exposed control group. The significance was confirmed with the PERMANOVA statistical test (*p*-value of 0.0001). Unlike the farm environment, nasopharynx samples from wastewater workers did not cluster with air samples from wastewater treatment plants. The difference in the microbial community of nasopharynx of swine workers and a control group suggest that swine workers are carriers of germs found in bioaerosols. *Conclusion:* Nasopharynx sampling and microbiota could be used as a proxy of air sampling for exposure assessment studies or for the determination of exposure markers in highly contaminated agricultural environments.

## 1. Introduction

The microbial flora of aerosols, referred to as bioaerosols, consists of a combination of viable and non-viable microorganisms (e.g., bacteria, fungi and viruses) and derived compounds of biological origin (e.g., animal and plant debris, endotoxins, exotoxins, and other microbial metabolites) [1,2,3]. Bioaerosols are ubiquitous in indoor and outdoor environments and are generated from various natural and/or anthropogenic sources. Composed of particles ranging in size from a few nanometers to 200 µm in diameter, bioaerosols remain suspended in the air for long periods of time and may travel many kilometers depending on the size of the particle [1,4,5,6,7,8]. Therefore, the dispersal of bioaerosols may impact the air quality of extensive areas that are far from the source and can create public health issues, due to the presence of highly diverse and dynamic microbial communities. Beyond affecting the time that particles remain suspended and the distances they travel, particle size plays a role in human diseases, as it dictates which pathway the particle follows in the respiratory tract after inhalation [9]. For example, particles with a size of 4 μm to 10 μm tend to get deposited in the upper airways, while larger particles may remain in the nasal cavity [10,11].

Bioaerosols can be a transmission vector for infectious diseases and are responsible for a variety of health problems, principally through inhalation [9,12,13,14,15]. Human exposure to bioaerosols is associated with a wide variety of acute and chronic diseases ranging from allergies, asthma, rhinitis, sinusitis and bronchitis, mostly due to occupational exposure [4,16,17,18,19]. However, health risks from bioaerosols also exist just from living in close proximity to an intensive source of airborne biological particles [20,21]. Additionally, other health problems linked to bioaerosols include fatigue, headache, mucous membrane irritation syndrome, nasal congestion, sore throat, and irritation of the nose and eyes [17,22,23].

The industrial environment is the main source of occupational health issues, due to the presence of raw organic materials, the prevalence of operations releasing harmful bioaerosols (e.g., mechanical operations such as wood planning, straw chopping, animal bedding, hay handling, and compost pile turning) and the eventual large amounts of bioaerosols present in confined spaces. For example, biowaste facilities are characterized by notable concentrations of bioaerosols, due to the intense microbial activity involved in waste degradation and the activities performed by workers [24,25,26,27]. Wastewater treatment plants (WWTPs) represent another environment where workers are subject to bioaerosol exposure, due to the steps required for the treatment of discharged municipal and industrial effluents [28,29]. Intensive animal farming practices in confined buildings that hold a large number of animals (e.g., pigs, poultry, cattle) are also associated with extreme exposure to airborne microbes. The variety of possible sources (e.g., animals, feces, feed, litter) present at farms leads to the emission of complex mixtures of biological particles [30,31,32,33,34,35]. Moreover, the dynamic nature of the microbial composition makes health risk evaluation complicated for farm workers and nearby residents [36,37]. Environmental hygienists continue to insist that insufficient exposure assessments are a primary reason for the absence of bioaerosol exposure limits and strategies to mitigate risk [35,38].

There are several challenges and limitations to measuring bioaerosol exposure. The type of sampler, time and duration of sampling, meteorological conditions [39], and geographical positions all affect bioaerosol sampling efficiency, making it difficult to compare studies and limiting collaboration efforts in bioaerosol exposure studies. An added challenge in terms of measuring microbial diversity is that culture-dependent analytical approaches recuperate the cultural/viable portion of bioaerosols exclusively. In contrast, high-throughput sequencing (HTS) methods are associated with a more in-depth characterization of the microbial content of a sample [26,27,40,41,42,43]. To overcome the aforementioned challenges, the scientific community that studies aerosols has identified the need to explore new alternatives and complementary approaches for assessing bioaerosol exposure, such as identifying markers that can help classify environments based on human health risks [44,45,46].

The upper respiratory tract, which includes the nasal cavity and the nasopharynx and is a primary pathway for inhaled air, is an important niche for transient environmental bacteria and for colonization. Previous studies have already used nasopharynx or nasal cavity samples to look for specific microorganisms, using culture-based approaches [11,47], or revealed the presence of bacterial resistance genes, using molecular biology approaches [33]. Recently, an HTS approach was used on samples from the anterior and posterior cavity of the nose to study the bacterial diversity of individuals [48]. However, because the microflora in the nasal cavity is dynamic and fluctuant [49], a region like the nasopharynx may represent a more long-term reservoir of inhaled bioaerosols. To the best of our knowledge, no study has ever used a 16S rRNA amplicon-based HTS approach to investigate the microbial diversity of nasopharyngeal flora, in order to assess occupational exposure. Microbiome studies that use upper respiratory tract tissue and focus on specific pathologies (such as asthma, chronic obstructive pulmonary disease and chronic rhinosinusitis) may underestimate the effect of occupational exposure on the microbiomes of the subjects examined in those studies. We hypothesis that the microbiome of patients with respiratory disease is most likely affected by exposure to their work environment in addition to disease, rather than to disease alone.

This study aims to contribute to the development of a new alternative method for assessing bioaerosol exposure, by linking the nasopharyngeal flora of pig farm workers to the bioaerosol microbial composition of their occupational environment, and to explore the role of bioaerosols in the nasopharyngeal microbial content. The 16S rRNA amplicon-based HTS approach was used to determine bacterial diversity, while qPCR was used to evaluate the presence of human pathogenic agents and bacterial resistance genes in bioaerosols and in nasopharyngeal samples from pig workers. Additionally, WWTPs were used as the industrial, low-dust, non-agricultural environment control to validate the microbial link between the bioaerosol content (air) and the nasopharynxes of workers. Although the experiment focused on pig farmers and the buildings that they work in, the results may have implications for a wider population of agricultural workers. Consequently, nasopharynx samples could be used as proxies for air samples in exposure assessment studies and for the determination of exposure markers in agricultural settings. In addition, the study advocates for the need of systematic bioaerosol exposure study when evaluating the nasopharyngeal microbiota.

## 2. Materials and Methods

### 2.1. Sampling Sites in Pig Buildings

Air samples were collected from eight confined pig buildings in Eastern Canada during fall/winter 2015. Inside each farm building, a sampling site was designated based on worker activities and bioaerosol exposure. The buildings visited were mechanically ventilated and contained between 800–1200 pigs each weighing between 90–120 kg. There were no obvious signs of illness affecting the animals.

### 2.2. WWTP Sampling Sites (As Non-Agricultural Environment Controls)

Air samples were collected from eight WWTPs in the province of Quebec, located in Eastern Canada, the during summer and winter seasons. Summer visits occurred between September 2015 and July 2016, and winter visits occurred between February 2015 and March 2016. Four sampling sites were chosen depending on the wastewater treatment process (screening, de-gritting/degreasing, settling tank, and bio-filtration), workers’ daily tasks, and the level of confinement of the space.

### 2.3. Air Sampling

A liquid cyclonic impactor Coriolis μ Biological Air Sampler (Bertin Corp., Rockville, MD, USA) was used for collecting air samples. The samplers were set at 300 L/min for 10 min (3 m^3^ of air per sample), placed within 1–2 m of the bioaerosol source and away from any turbulent air flow (e.g., away from building exhaust fans). Fifteen milliliters of a phosphate buffer saline (PBS) solution (0.0067 M, pH 7.4, Lonza, Walkersville, MD, USA) were used as the collecting solution.

### 2.4. Nasopharynx Sampling

Nasopharyngeal samples were taken from 29 controls (university students and staff never exposed to animal farms), 25 pig farmers and 12 WWTP workers between the fall of 2015 and spring 2017. All controls, pig farmers, and WWTP workers were non-smokers and none were taking antibiotics. The protocol was approved by the Ethics Committee of the Institut universitaire de cardiologie et de pneumologie de Québec (CER21221). Nasopharyngeal samples were collected by a nurse using swabs (Puritan^®^ HydraFlock^®^ Collection Devices, Guilford, MA, USA) with a dry secure transport system. Briefly, a swab was inserted into the nose until a resistance was felt and then turned a few times before it was removed. Samples were transported to the laboratory at 4 °C.

### 2.5. Pre-DNA Extraction Treatment

From the 15 mL collecting solution of the Coriolis µ Biological Air Sampler (Bertin Corp.), a 1.5 mL aliquot was centrifuged for 10 min at 14,000× *g* (J. Pilote protocol = 3 mL aliquot, 10 min, 21,000× *g*). The supernatant was discarded and the pellets were kept at −20 °C until DNA extraction. Likewise, the tips of the swabs were cut and vortexed thoroughly in 1 mL PBS (Lonza) and discarded. Suspensions were then centrifuged for 10 min at 21,000× *g*, the supernatants were discarded and the pellets were stored at −20 °C until DNA extraction.

### 2.6. DNA Extraction

Total genomic DNA from air and nasopharyngeal samples was extracted with a PowerLyser^®^ Powersoil Isolation DNA kit (MO BIO Laboratories, Carlsbad, CA, USA) following the manufacturer’s instructions. DNA samples were stored at −20 °C until subsequent analyses.

### 2.7. MiSeq Illumina^®^ Sequencing

The amplification of targeted genes, equimolar pooling, and sequencing were performed at the Plateforme d’analyses génomiques (IBIS, Université Laval, Québec, QC, Canada). The 16S rRNA V6-V8 region was amplified using the sequence-specific regions described in Comeau et al. 2011 using a two-step dual-indexed PCR approach, specifically designed for Illumina^®^ instruments, San Diego, CA, USA [50]. The gene-specific sequence was first fused to the Illumina^®^ TruSeq sequencing primers and PCR was carried out in a total volume of 25 µL containing 1 × Q5 buffer (NEB, Ipswish, MA, USA), 0.25 µM of each primer, 200 µM of each of the dNTPs, 1 U of Q5 High-Fidelity DNA polymerase (NEB) and 1 µL of template DNA. PCR thermoprotocol began with an initial denaturation at 98 °C for 30 s followed by 35 cycles of denaturation at 98 °C for 10 s, annealing at 55 °C for 10 s, extension at 72 °C for 30 s and a final extension at 72 °C for 2 min. The PCR reaction was purified using the Axygen PCR cleanup kit (Axygen^®^, Waltham, MA, USA). The quality of the purified PCR product was checked on a 1% agarose gel. A fifty to 100-fold dilution of the purified product was used as a template for a second round of PCR in order to add barcodes (dual-indexed) and for missing sequences required for Illumina sequencing. The thermoprotocol for the second PCR was identical to the first one but with 12 cycles. PCR reactions were purified again in the same way as above, checked for quality on a DNA7500 Bioanalyzer chip (Agilent^®^, Santa Clara, CA, USA) and then quantified spectrophotometrically with the Nanodrop^®^ 1000 (Thermo Fisher Scientific, Waltham, MA, USA). Barcoded amplicons were pooled in equimolar concentrations for sequencing on the Illumina^®^ MiSeq machine. The oligonucleotide sequences that were used for PCR amplification are presented in Table 1.

### 2.8. Bioinformatics

Briefly, after de-multiplexing the raw FASTQ files, the reads generated from the paired end sequencing were combined using the *make.contigs* script from mothur [51]. Quality filtering was also performed with mothur, using the *screen.seqs* script to discard homopolymers, reads with ambiguous sequences, and reads with suspiciously short lengths. Similar sequences were gathered together in order to reduce the computational burden, and the number of copies of the same sequence was displayed to monitor the abundance of each sequence. This de-replication step was performed with VSEARCH [52]. The sequences were then aligned with the bacterial reference SILVA core alignment using the QIIME script *align_seqs.py* [53]. Operational taxonomic units (OTUs), with a 97% similarity cut-off, were clustered using the UPARSE method implemented in VSEARCH. UCHIME was used to identify and remove the chimeric sequences [54]. QIIME was used to assign taxonomy to OTUs based on the SILVA database reference training dataset for taxonomic assignment and to generate an OTU table. A metadata-mapping file was produced that includes information about air and nasopharyngeal samples. The microbial diversity analyses, including statistical analyses, conducted in this study, were achieved using QIIME plugins in version 1.9.0 as described in QIIME scripts (http://qiime.org/scripts/). The names of the scripts used are mentioned in the results section of each analysis.

### 2.9. Quantification of Human Pathogens and Resistance Genes in the Nasopharynx

PCR was performed with CFX-96 and CFX-384 Touch™ Real-Time PCR Detection Systems (Bio-Rad Laboratories, Mississauga, ON, Canada) to evaluate the presence of six human pathogens (*Clostridium difficile*, *Listeria monocytogenes*, *Mycobacterium avium*, *Salmonella* spp., *Staphylococcus aureus*, and methicillin-resistant *Staphylococcus aureus* (MRSA)), and antibiotic and metal resistance genes (cephalosporin, colistin, zinc). The PCR mixture contained 2 μL of DNA template, 150–300 nM for each primer, 100–125 nM probe and 10 μL of 2 × iQ™ supermix or iQ™ SYBR^®^ Green supermix (Bio-Rad Laboratories) in a 20 μL reaction mixture. The results were analyzed using Bio-Rad CFX Manager software, version 3.1 (Bio-Rad Laboratories). Positive control and standard curves ranging from 1 × 10^6^ to one copy of the targeted genes were used for each protocol using genomic DNA or synthetic genes as templates (Integrated DNA Technologies, Coralville, IA, USA). Negative controls were included in the plates as NTC (non-template controls). All primers and probes were purchased from Integrated DNA Technologies. The primers, probes, hybridization temperatures, amplicon sizes and original references for all targeted genes are listed in Pilote et al. 2019 [55].

### 2.10. Statistical Analyses

For alpha diversity measures, the normality was verified using the D’Agostino and Pearson omnibus normality test. The assumption of data normality was not fulfilled. Non-parametric Mann-Whitney *U* tests (two-tailed) were performed to highlight that there are significant differences in diversity measures between the groups of samples. A *p*-value ≤0.05 was considered statistically significant. All of the results were analyzed using the software GraphPad Prism 5.03 (GraphPad Software, Inc., San Diego, CA, USA). To determine the statistical significance of the variation in the observed microbial community composition with multivariate analyses (PCoA), a PERMANOVA test was performed on the Unweighted UniFrac matrix. The *compare_categories.py* QIIME script was used to generate the statistical results. Because PERMANOVA is a non-parametric test, significance is determined through permutations. In this case, 999 permutations were used. A *p*-value ≤0.05 was considered to be statistically significant. Detailed information about the performance of the test is presented in the multivariate section of the results. The non-parametric Mann-Whitney *U* test was used to ascertain whether or not differences in OTU abundances were statistically significant between the controls and pig farmers. To test OTU differential abundance, the null hypothesis was that the populations that the two groups of samples were collected from have equal means. The range of p-values obtained for the 30 most differentially abundant OTUs between the control samples and the pig farmer samples are presented in the differential abundance section of the results.

### 2.11. Experimental Controls

This study used both positive and negative controls. The negative controls include unused swabs that underwent the same extraction protocol as the swabs collected from the subjects of this study. A PCR amplification targeting the 16S rRNA genes allowed us to confirm the very low biomass of the negative controls compared to the nasopharyngeal swabs from the pig workers and non-exposed controls. For this reason, negative controls did not pass the next step of Illumina HTS. Additional negative controls consisted of outdoor air samples that were taken outside the pig buildings sampled in this study. These samples showed enough concentration of bacterial biomass with the PCR amplification. Thus, outdoor negative controls were sequenced. However, the number of reads and subsequent OTU clustering was low compared to the indoor air samples. During the rarefaction step, the negative controls were not included in the analyses due to a low number of sequences. The goal is to have a number of sequences per sample deep enough to cover most of the bacterial diversity. Positive controls consisted of a mock community containing equal concentrations of 20 bacteria purchased from ATCC (20 Strain Even Mix Genomic Material ATCC^®^ MSA-1002^TM^). Sequencing of the mock community showed a taxonomic profile resembling the expected microbial community, but with different relative abundances.

## 3. Results

### 3.1. Summary of Data Processing

In total, 8 air samples from pig buildings, 25 nasopharynx samples from farmers and 29 nasopharynx samples from the non-exposed control group resulted in 2,942,265 sequences (air samples = 39,425; farmers = 1,900,800, controls = 1,002,040). Following quality filtering and the discarding of singletons, 1,425,981 unique sequences clustered into 6060 OTUs. Representing the non-agricultural control environment (WWTPs), 1,190,166 sequences came from 8 air samples (98,285) and 12 nasopharynx samples (1,091,881) from plant workers. After quality filtering and the removal of singletons, 51,969 unique sequences clustered into 2188 OTUs.

### 3.2. Alpha Diversity

#### 3.2.1. Rarefaction Curves (Number of Observed OTUs)

**Figure 1:**
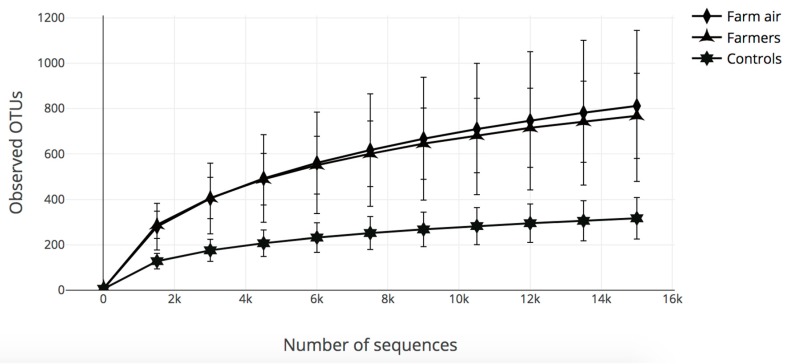
Rarefaction curves obtained from the number of observed operational taxonomic units (OTUs) and the sequences per sample for air samples from pig farms and nasopharynx samples from pig farmers and non-exposed controls. The plateau of the curves started around 2000 sequences.

A rarefaction analysis was performed to validate the sequencing depth and to confirm the effective sampling of the microbial diversity using the *alpha_rarefaction.py* QIIME script. The lowest-depth sample parameter was used for the rarefaction analyses, allowing equal numbers of sequences for all samples. Therefore, the samples with a sequencing depth lower than the reference sample were excluded from the analyses. The higher the sequencing depth, the more likely that the full diversity coverage is attained. The sequencing depth was 15,000 sequences for all the groups of samples: The air samples from pig buildings, the nasopharyngeal samples of pig farmers and non-exposed controls. The points shown in Figure 1 were calculated as follows: Ten values from 10 to 15,000 analyzed sequences were randomly selected. For each of these values, the corresponding number of OTUs observed, was noted for all of the samples. Then, the average number of OTUs observed, plus or minus one standard deviation, were calculated for each of the ten values. The samples were divided into three groups: *Air from pig buildings*, *Pig farmers* and *Non-exposed controls*. The slope of the curves shows sufficient sequencing depth and good bacterial coverage in all samples. Moreover, pig farmers and air samples showed the highest average number of OTUs compared to non-exposed controls.

#### 3.2.2. Richness Estimates and Diversity Measures

**Figure 2:**
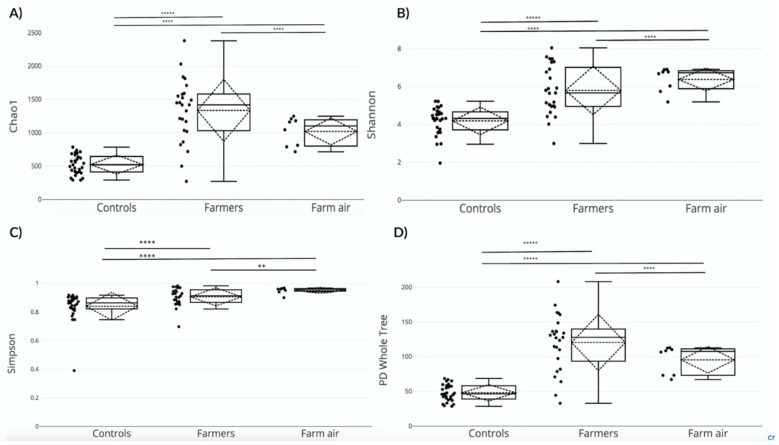
Alpha diversity analyses comparing nasopharynx samples of pig farmers and non-exposed controls and air samples from pig buildings. Analyses include (**A**) Chao1, (**B**) Shannon, (**C**) Simpson, and (**D**) phylogenetic diversity (PD) Whole Tree. The boxplots represent the maximum, minimum value, first and third quartile values, and the median value. The mean values with the standard deviation are represented by the dotted lines. The asterisks (*) indicate the statistical significance of the Mann-Whitney *U* tests (ns *p* > 0.05; ** *p* ≤ 0.01; **** *p* ≤ 0.0001).

Four indexes were used to measure alpha diversity using the *alpha_diversity.py* script: Chao1 richness estimator (the higher the number of OTUs in a sample, the higher the value of the Chao1 index). For a more detailed explanation about richness estimate calculation, please refer to http://chao.stat.nthu.edu.tw/wordpress/paper/119.pdf. In Shannon and Simpson diversity measures, richness is combined with abundance to obtain an evenness measure. Simpson values are bounded between 0 and 1, where 1 represents the most diverse case. Shannon values are bounded between 0 and 10, where 10 represent the highest diversity) and phylogenetic diversity (PD) whole tree (quantitative measure of phylogenetic diversity; the higher the value, the higher the diversity; no limit value). The nasopharynx samples from pig farmers consistently showed the highest richness estimates and diversity measure values, whereas non-exposed controls displayed the lowest values (Figure 2A–D). The difference between the two groups of samples was significant (Chao1 *p* = 0.000001; Shannon *p* = 0.000009; Simpson *p* = 0.00004; PD Whole Tree *p* = 0.000006). The richness estimates and diversity measures in the air samples were nearly as high as the pig farmer nasopharynx samples, although the measures from the pig farmer samples were statistically higher (Chao1 *p* = 0.00001; Shannon *p* = 0.0002; Simpson *p* = 0.001; PD Whole Tree *p* = 0.00002). The difference between the air samples from pig buildings and non-exposed controls (nasopharynx) was significant as well (Chao1 *p* = 0.00005; Shannon *p* = 0.00001; Simpson *p* = 0.00007; PD Whole Tree *p* = 0.000009).

#### 3.2.3. Beta Diversity

**Figure 3:**
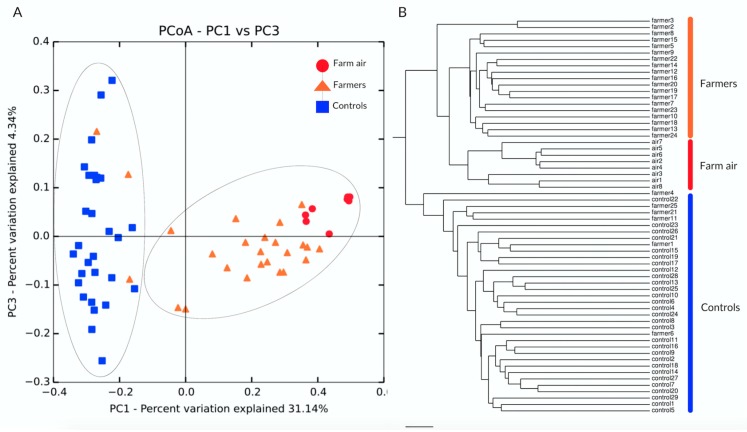
Pairwise sample dissimilarity compares the nasopharynxes of farmers and of non-exposed controls and air from pig buildings. The pairwise distances were calculated using the weighted UniFrac distance metric. (**A**) Principal coordinate analysis plot showing distances in the composition of the microbiota from three groups of samples. (**B**) Phylogram showing sample clustering using the unweighted pair group method with arithmetic mean. The clustering (air from pig buildings and pig farmers together, versus the non-exposed controls) was statistically significant as confirmed by the PERMANOVA test (*p*-value = 0.0001).

An ecological analysis was conducted to reveal the variation in the community composition between the three sample groups (nasopharynx of pig farmers and non-exposed controls and air from pig farms). The weighted UniFrac distance metric was used to calculate the pairwise distances between samples using the *beta_diversity.py* script. The distance matrix was then transformed into coordinates using the *principal_coordinates.py* script and inter-samples distances were represented in a two-dimensional (2D) space using ordination. The samples closer to one another were more similar than those ordinated further apart. The principal coordinate analysis (PCoA) was used to visualize bacterial community variation (*make_2D_plots.py*). Figure 3A shows the two principal coordinate axes capturing a total of 35.48% of the variation observed. A distinct clustering of pig farmers, non-exposed controls, and air samples from pig buildings is also illustrated in that figure. The profiles of pig farmers were more similar to the profiles of air samples than to the profiles of non-exposed controls. The distinct clustering was confirmed by the per-mutational multivariate analyses of variance (PERMANOVA *p* = 0.0001). The same statistical test was used to confirm the non-significant clustering of air and pig farmer (nasopharynx) samples, as the test showed a non-significant difference (PERMANOVA *p* = 0.08). Interestingly, air samples from the pig buildings seemed to display less dispersion amongst its individuals than the farmers and non-exposed groups, indicating a more homogenous bacterial community structure. We used a phylogram that displays sample clustering, using the unweighted pair group method, with arithmetic mean to confirm the sample clustering observed with the PCoA analyses (Figure 3B).

### 3.3. Taxonomy Identification

#### 3.3.1. Phylum Profiles

**Figure 4:**
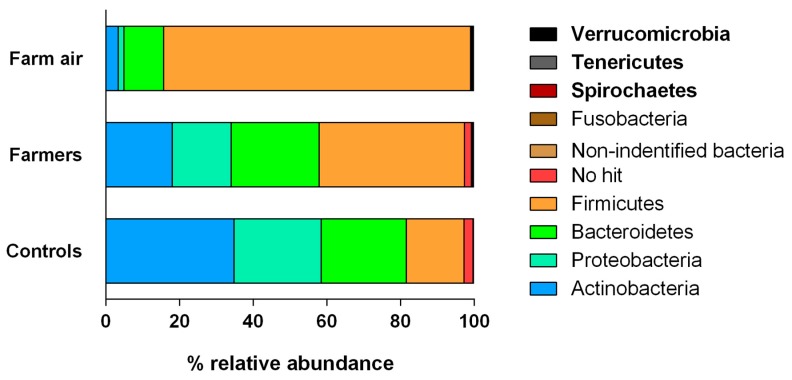
Taxonomic profiles showing the relative abundance of each bacterial phylum across nasopharyngeal flora samples from farmers, non-exposed controls and air samples from pig buildings. Phyla names written in bold type were specific to farmers and air from pig buildings.

Given the observed difference in the number of bacterial OTUs, evenness, and evolutionary distance (alpha diversity) and in the bacterial community composition (beta diversity) in samples of the nasopharyngeal flora of farmers and non-exposed individuals and bioaerosols, collected in pig buildings, the next step was to reveal the taxonomic profiles of the three groups. Figure 4 shows the taxonomic distribution of the bacterial phyla across the three groups of samples. Overall, Actinobacteria, Proteobacteria, Bacteriotedes, and Firmicutes dominated the three profiles, representing more than 95% of the taxonomic abundance. However, major differences distinguished the pig farmer samples from the non-exposed controls. In the latter, Actinobacteria and Proteobacteria were the most abundant phyla (relative abundances of 35%, and 24%, respectively). However, in farmers, Firmicutes and Bacteriotedes were the most dominant phyla with relative abundances of 40%, and 24%, respectively. Consistent with the previous analyses, air samples from pig farms had different relative abundances values, but comparable profiles (with the same conclusions) to the nasopharyngeal flora of farmers, with a dominance of Firmicutes (83%), followed by Bacteriotedes (11%). Actinobacteria, and Proteobacteria had a relative abundance of less than 5% in bioaerosol samples. Notably, Spirochaetes, Tenericutes, and Verrumicrobia were detected only in farmers and the air from pig buildings.

#### 3.3.2. Class Profiles

**Figure 5:**
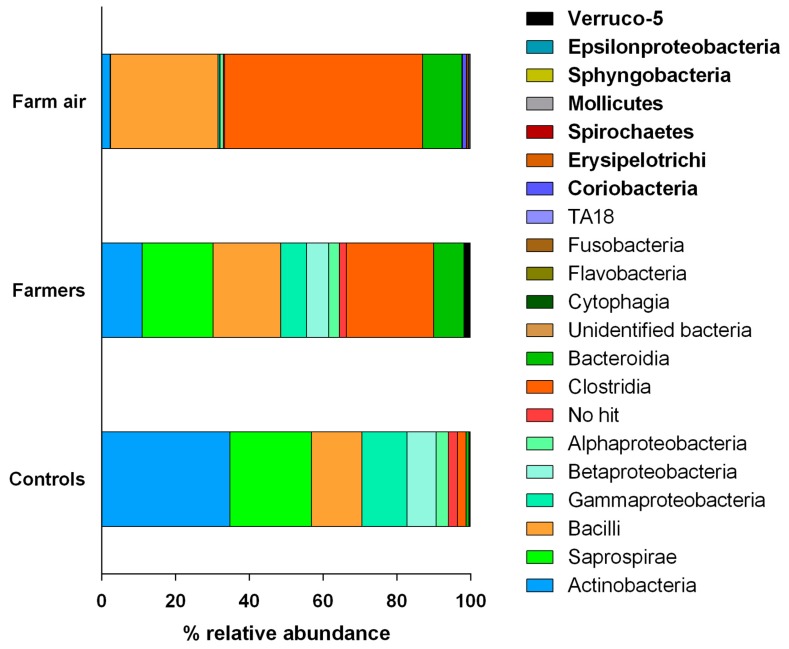
Taxonomic profile showing the relative abundance of each bacterial class across nasopharyngeal flora samples from pig farmers, non-exposed controls and air samples from pig buildings. Taxa written in bold type were specific to farmers and air from pig buildings.

The relative abundance of taxa was more thoroughly analyzed by examining the most abundant bacterial classes across the three groups of samples (Figure 5). Similar to the phyla profiles, the class profiles showed notable differences between non-exposed controls and farmers/air from pig buildings. In the former, Actinobacteria (39%), Saprospirae (23%), Bacilli (11%), Gammaproteobacteria (10%) and Betaproteobacteria (8%) represented more than 90% of the taxonomic profile. However, the profile from farmers was more evenly distributed. Clostridia had the highest relative abundance (24%) followed by Saprospirae (19%), Bacilli (18%) and Actinobacteria (11%). Unlike the non-exposed control group, Gammaproteobacteria and Betaproteobacteria represented less than 10% of the profile, whereas Bacteroidia represented 10% of the relative abundance. In the non-exposed control group, Bacteroidia represented 0.7% of the taxonomic profile. In air samples, Clostridia, Bacilli and Bacteroidia dominated the profile representing more than 90% of the relative abundance, thus confirming the previous observations about the similarity between the flora from pig farmers and air samples. Interestingly, the presence of Coriobacteria, Erysipelotrichi, Spichaetes, Mollicutes, Sphyngobacteria, Epsilonproteobacteria, and Verruco-5 was exclusive to samples from the nasopharynx of pig farmers and sampled bioaerosols.

#### 3.3.3. Differential Abundance of Species

**Figure 6:**
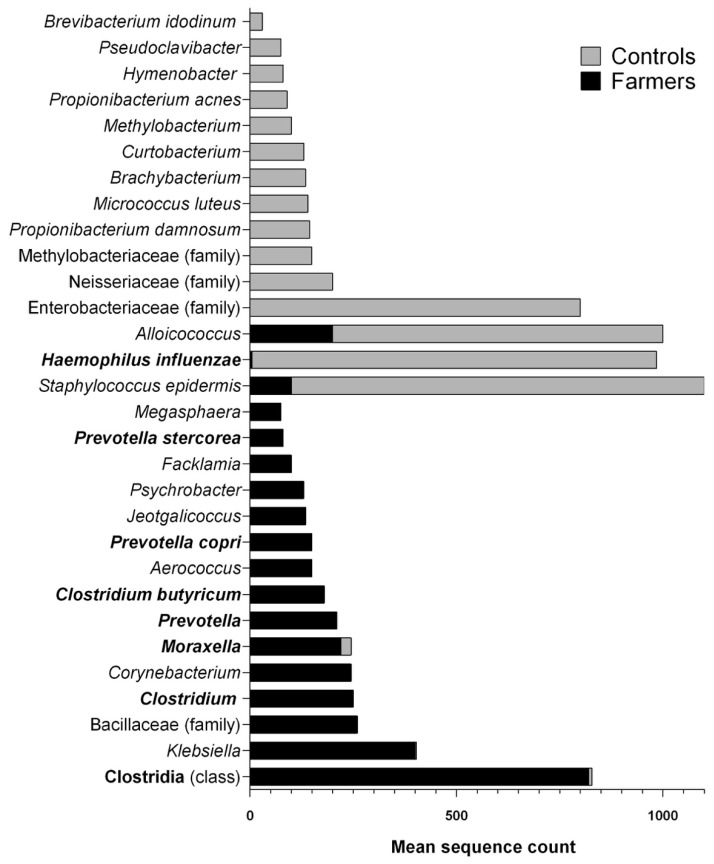
Taxa identified to highest possible taxonomic level with statistically significant differential abundances across pig farmers and non-exposed controls. From the bottom to the top: The first 15 taxa were the most abundant in samples from farmers and the last 15 were more abundant in non-exposed controls. The taxa written in bold type affect human health.

A non-parametric Mann-Whitney *U* test, was used to analyze count data and determine the species most significantly associated with farming. The test compares OTU frequencies in groups of samples and ascertains if there are statistically different OTU abundances between the two groups of samples. The Mann-Whitney *U* test uses absolute data counts rather than relative abundances. More specifically, the output of the test contains the test statistic, the p-value corrected for multiple comparisons, and a mean count for each OTU in the given sample group. This test was used following instructions from the *group_significance.py* QIIME script. The thirty taxa (identified to the species or genera) with the greatest significant differences in counts between samples from pig farmers and non-exposed controls are presented in Figure 6. However, to better visualize and emphasize the most striking cases of differential abundance, the list is not exhaustive. The complete results output of differential abundance is presented in Additional file 1 (Supplementary Materials). It represents the results of the Mann-Whitney *U* test to determine the statistical differential abundance of taxa in nasopharynx of workers and non-exposed controls. The test was applied to sequences using qiime script (*group_significance.py*) with the Mann-Whitney *U* test option. The taxonomy represent bacteria from the nasopharynx samples. Notably, some taxa were identified only to class or family, as those were the highest levels of identification possible using the SILVA database. *p*-values were corrected for multiple comparisons using the Bonferroni correction. Values ranged from 0.0000007 to 0.0001 for the 15 differentially abundant taxa, from pig farmer samples, and from 0.000002 to 0.0005 for the 15 differentially abundant taxa, from non-exposed controls. The most notable imbalance was observed for the class Clostridia with a mean count of more than 800 sequences in pig farmer samples and less than 10 sequences in the non-exposed controls. *Staphylococcus epidermis* was present with a mean count of 1000 sequences in non-exposed individuals and less than 100 sequences in pig farmers. Other important examples related to human health include, the greater differential abundance of *Haemophilus influenzae* in non-exposed controls (950 sequences in non-exposed control samples vs. 5 in samples from pig farmers), and the differential abundance of *Klebsiella* in samples from pig farmers (400 sequences in pig farmer samples vs. 3 in non-exposed controls).

#### 3.3.4. Non-Agricultural Low-Dust Industrial Control Environment

**Figure 7:**
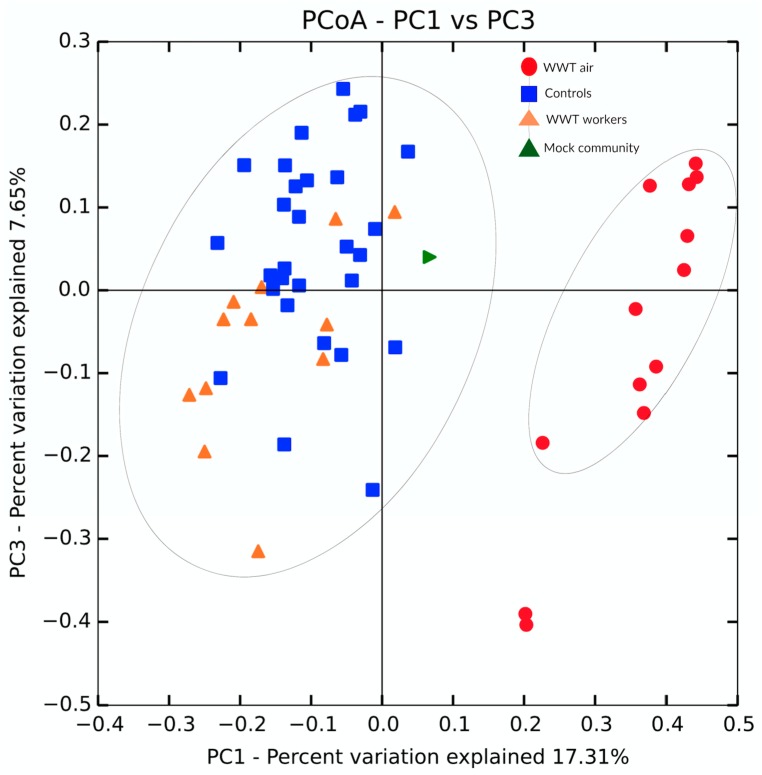
Principal coordinate analysis plot. The plot shows the distances for the microbiota of three groups of samples: nasopharynx samples from wastewater workers and from non-exposed controls and bioaerosols from Wastewater Treatment Plants (WWTPs). The pairwise distances were calculated using the weighted UniFrac distance metric.

A non-agricultural low-dust control environment (WWTPs) was used as a control to validate the link between the microbial composition of nasopharyngeal flora of exposed workers and that of bioaerosols released in the workplace. The nasopharynx samples of the non-exposed controls (subjects not previously exposed to any animal farm) were again used for comparison with the nasopharynx samples from wastewater workers and air samples from WWTPs. The distances between the groups of samples were compared and visualized using the PCoA approach. Similar to the pig farm environment, the pairwise distances were calculated using the weighted UniFrac distance metric. Figure 7 shows the two principal coordinate axes capturing a total of 24.96% of the variation observed. Unlike the farm environment, the nasopharynx samples from wastewater workers did not cluster with air samples from WWTPs. In fact, nasopharyngeal flora of wastewater workers and non-exposed controls had similar microbial compositions. The difference between air and nasopharynx samples (controls and wastewater workers) was statistically significant (PERMANOVA *p* = 0.0006). As shown in Figure 7, the difference between non-exposed controls and wastewater workers was not significant (PERMANOVA *p* = 0.1).

#### 3.3.5. Human Pathogens and Resistance Genes in the Nasopharynxes of Pig Farmers and Non-Exposed Controls

The presence of human pathogens was investigated in the nasopharynx of pig farmers. As noted in Table 2, all of the pathogens were more frequently detected in the pig farmer nasopharynx samples, compared to non-exposed controls, with the exception of *Salmonella* spp. Striking examples include, MRSA and *Clostridium difficile*, which were present in the nasopharyngeal flora of 60%, and 12% of pig farmers, respectively. They were found in 10%, and 0% of the non-exposed controls, respectively. *Listeria monocytogenes* was detected in 3% of non-exposed controls and in 24% of pig worker samples. *Mycobacterium avium* was not detected in the nasopharynx samples of either group. Likewise, antibiotic and zinc resistance genes were present at a higher frequency among pig farmers compared to non-exposed controls. Moreover, cephalosporin, and colistin resistance genes were exclusively detected in the nasopharyngeal flora of pig farmers.

## 4. Discussion

Given the many potential microbial sources, animal farmers inhale a variety of aerosolized bacteria that impact their health [18,56,57]. In this study, the bacterial populations in bioaerosols from pig buildings were compared to those of the nasopharyngeal flora of farmers using bioinformatics tools to determine if nasopharynx sampling could be used as a proxy for air sampling in exposure assessment studies. Systemic microbial ecology analyses led to unequivocal results with identical conclusions throughout the analyses.

The alpha diversity of bacterial species in the air from pig buildings and the nasopharyngeal flora of farmers were not statistically different. The evaluation of species diversity was introduced by Whittaker and defined as the number of species and their proportional abundance within one sampling site [58]. There are different ways to measure alpha diversity and an extensive list of indexes has been presented by Magurran and McGill [59]. In addition to the usual Chao1 richness estimates and Shannon/Simpson diversity measures [60,61,62,63], PD Whole Tree was also used to analyze the alpha diversity in samples in this study. PD stands for phylogenetic diversity and is defined as the minimum length of all phylogenetic branches required to span a given set of taxa on the phylogenetic tree [64]. All four of the alpha diversity measures revealed greater bacterial richness and diversity in the nasopharyngeal samples from pig farmers compared to non-exposed individuals. The observed similarity between bioaerosols from pig buildings and the nasopharyngeal flora from farmers is indicative of occupational exposure and, consequently, a transient presence and/or possible colonization of the upper respiratory tract regions by environmental bacteria. These findings are even more interesting given that the majority of pig farmers recruited for this study do not work in the eight pig buildings selected for air analysis. This suggests that airborne bacteria associated with pig buildings can take over the microbiota in farmers’ nasopharynxes. The establishment of this “new” microbial community could represent a microbial signature for the nasopharynx of pig farmers. Also, a higher prevalence of viruses in the nasopharynxes of farmers compared to the non-exposed control group could play a role in the increased alpha-diversity [65].

Beta diversity analyses revealed that long-term exposure, such as occupational exposure to bioaerosols in the air of pig buildings, appeared to modify the nasopharyngeal microbiota of farmers. Common approaches to evaluating changes in the community composition (beta diversity) rely on the creation of a (dis)similarity matrix to calculate the distance between samples. Dis(similarity) matrices may be calculated using different methods depending on the type of dataset, analyses, and the objectives of the study, as some metrics are more suitable than others [66,67,68,69]. The UniFrac distance metric was used as efficacy was proven with 16S rRNA bacterial genes [70]. In addition, PCoA coupled with PERMANOVA offers a robust statistical significance of sample grouping using distance matrices. This non-parametric multivariate analysis of variance separates the distance matrix into sources of variation to describe the robustness and significance of a variable in explaining the variations observed between samples. It is based on the ANOVA experimental design but analyzes the variance and determines the significance by permutations, as it is a non-parametric test [71]. Whereas, ANOVA/MANOVA assumes normal distributions and a Euclidean distance, PERMANOVA can be used with any distance measure. The two analyses led to the same conclusions for this study. Therefore their usefulness when used together as a tool to visualize and evaluate sample clustering was confirmed. The distinct clusters formed between the combination of pig farmers, and the air from pig buildings, and non-exposed individuals, is clearly linked to a strong divergence in the nasopharyngeal microbiota of farmers compared to other non-exposed individuals. Mechanical deposition of <100 µm diameter inhaled particles on nasopharyngeal surfaces by inertial impaction [72], represents a continuous source of environmental bacteria to the nasopharynx. This continuous source of bacterial exposure may therefore be responsible for the establishment of a reservoir of bacteria reflecting long-term exposure (e.g., occupational exposure). A thorough understanding of the established bacterial community may then lead to a better evaluation of the risks associated with an environment. For example, domestic animals share some of their microbiota with their human cohabitants, supposedly through frequent and direct contact [73,74]. Song et al., (2013) mention that airborne microbiota plays an important role in microbial transfer to the human upper respiratory tract. Ten thousand litres of air are inhaled daily and the bioaerosols in the air may have an effect on the human nasal microbial community [75]. Finally, as different farm animals are associated with different microbiota, the normal nasopharyngeal flora of farmers may be differently disturbed. In other words, farmers working with different animals may have a different disturbances of their natural nasopharyngeal flora. Therefore, the microbial fingerprint of the nasopharynx may be directly linked to a specific type of farming environment and the potential long-term health effects on farmers.

The high abundance of Firmicutes and Bacteriotedes in pig buildings has been shown [76,77,78]. Interestingly, the results of this study are consistent with the literature, but with the added information indicating that, these same phyla colonize the nasopharynxes of farmers. More particularly, other studies have previously shown Clostridia to be the most abundant class of bacteria in bioaerosols from pig buildings [30]. This study not only confirmed the abundance of Clostridia in air samples, but also that this class was the dominant class found in nasopharynx samples, while it was practically absent in non-exposed individuals. *Clostridium spp*., identified as differentially abundant in the farmer nasopharyngeal samples in the present study, comprise potentially pathogenic species [79]. Specifically, *Clostridium butyricum*, also differentially abundant in samples from farmers, has been identified as an emerging pathogen by public health authorities. Some *C. butyricum* pathogenic strains were associated with the occurrence of necrotizing enterocolitis, a bowel disease [80]. In addition, *Prevotella* spp., which was strikingly more abundant in nasopharynx samples, is a well-known agent involved in upper respiratory tract infections [81,82,83]. *Moraxella* is another genus identified by the differential abundance analyses as being predominant in the nasopharynxes of farmers. Like for other taxa identified in this study, strains of *Moraxella* spp. were previously detected as airborne bacteria in pig buildings [84]. The rate of colonization of *Moraxella* spp. in healthy adult populations is around 3% [85]. Species of this genus are opportunistic pathogens responsible for upper and lower respiratory tract infections [86]. Taxa identified exclusively in nasopharyngeal samples and air samples from pig buildings could be candidates for new markers to assess exposure to bioaerosols in pig farming environments.

Although, Actinobacteria are most abundant in the non-exposed controls, no pathogen was identified in the most abundant taxa presented in Figure 6 (except Haemophilus influenza). An example of the most abundant Actinobacteria in non-exposed control is *Micrococcus Luteus* that was differentially more abundant in controls compared to farmers. Another example of Firmicutes is *Staphylococcus epidermis* that was more abundant in the controls compared to the farmers.

The respiratory health of farmers has been of great interest for testing the hygiene hypothesis that stipulates that exposure to microbes from intensive farming during early life could be beneficial to health in adulthood [87,88]. However, the acceptance of the hygiene hypothesis is not unanimous in the scientific community [89]. In this study, taxa identified as differentially abundant among farmers could hypothetically play a role in the prevention of allergy and the development of atopic diseases. Indeed, some bacteria identified through this investigation (e.g., *Pedobacter*, *Pelomonas*, and *Megasphaera*) have been linked to healthy respiratory conditions [90].

A recent study, conducted by Kraemer and colleagues, also found a distinct clustering between samples from the nasal cavities of pig farmers and air samples from their workplace, when compared to the nasal cavities of non-exposed individuals [48]. Moreover, samples from the nasal cavities of cow farmers clustered separately from pig worker samples and air samples from pig buildings [48]. Although the nasal cavity is a more transient environment for environmental bacteria than the nasopharynx, it confirms the hypothesis of a microbial fingerprint specific to the farming environment. It supports the idea of creating a worldwide database, that lists potential markers specific to certain environments, and to the nasopharynxes of the people working in them. This database could represent an important asset for associating bioaerosol exposure with health problems [91].

The lack of a correlation between the nasopharynx of wastewater workers and bioaerosols from WWTPs could be explained by the nature and duration of exposure. Workers wear personal protective devices (e.g., masks) at certain working sites (e.g., biofiltration), which may affect the establishment of a ‘new’ environmental microflora. Supporting this idea, the most abundant bacteria, shared by non-exposed controls and wastewater workers, are naturally occurring skin bacteria like *Propionibacterium*, *Corynebacterium*, *Staphylococcus*, *Streptococcus*, and *Cutibacterium*. These taxa were not present (relative abundance less than 1%) in air samples from WWTPs (data not shown). Farmers do not usually wear respiratory protection and, moreover, they often live in the farming environment (e.g., in a house located near farms) and are consequently continuously exposed to the microbes generated from farming activities (occupational and residential exposures). Therefore, the exposure that farmers are subjected to is more likely to modify their natural nasopharyngeal flora. The agricultural/non-agricultural hypothesis presented in this work regarding the nasopharyngeal flora of exposed workers should be validated in other agricultural and industrial environments.

Recent studies of the microbiome of the upper respiratory tract may have underestimated the influence of occupational exposure when considering the effect of a particular disease on the natural flora of upper respiratory tract tissue [92,93,94]. The fact that the work environment may affect the natural flora of an exposed person on a long-term scale is a crucial consideration when he/she becomes a patient whose upper respiratory tract microbiota is the target of the disease. The results obtained in this work emphasize the importance of considering the environment of the nasopharyngeal flora of exposed workers, who are or could become patients suffering from chronic respiratory diseases. In the same way that recent advances in methods for identifying microbes has helped implicate the upper respiratory tract microbiome in inflammatory respiratory diseases, evaluating bioaerosol exposure can help us support the roles of resident microbes in both healthy and diseased tissues.

Specific human pathogens and antibiotic and zinc resistance genes were detected in the nasopharynxes of pig farm workers as well as in bioaerosols of pig buildings [55]. For bacterial diversity analyses, the qPCR approach supports the use of the nasopharynx as an alternative to air sampling. The presence of zinc and antibiotic resistance genes, in the nasopharynxes of farmers, is explained by the use of zinc and antibiotics in animal farming (for therapeutic or sub-therapeutic use, such as for growth promotion) and implies possible human health risks. Extensive use of zinc in pig feed is responsible for the proliferation of zinc-resistant bacterial communities at farms [95]. Cephalosporin is a commonly used antimicrobial drug in human infections and the spread of its resistance constitutes part of the antibiotic resistance crisis [96]. Finally, the *mcr-1* gene was found in the nasopharynxes of half of the pig farmers in this study, although the use of colistin is extremely regulated in North America and limited to multi-drug resistant microbes [97].

Future studies should include detailed health information on the sampled individuals to investigate the nasopharynx microbiota associated with certain occupational health problems. Additionally, a longitudinal study of the bacterial diversity, in the nasopharynxes of farmers and in bioaerosols from pig buildings, could unveil a long-term variation in microbial content. Finally, information about the diet of exposed human (or animal) and antibiotic use could be added to the analyses as important factors influencing the microbiota.

## 5. Conclusions

This is the first study to link the nasopharyngeal flora of exposed humans with the source of the exposure in an agricultural setting, using bacterial diversity analyses and the detection of specific pathogens and resistance genes. The results suggest that workers are carriers of bioaerosol-associated bacteria and that nasopharynx sampling could be used as a proxy for air sampling in exposure assessment studies. Furthermore, pig farmers are also carriers of specific human pathogens and resistance genes that are worthy of health concerns. However, the present study did not confirm the use of nasopharynx sampling as an alternative for air sampling in non-agricultural environments (wastewater treatment). Thus, we conclude that the nasopharynx could be a reservoir for environmental bacteria that reflects the long-term exposure to bioaerosols in agricultural settings.

## Figures and Tables

**Table 1:** Primers used for PCR amplification of the 16S rRNA gene.

Primers ^a^	Sequences 5′ to 3′
First-PCR primers	Forward: 5′-ACACTCTTTCCCTACACGACGCTCTTCCGATCTACGCGHNRACCTTACC-3′
	Reverse: 5′-GTGACTGGAGTTCAGACGTGTGCTCTTCCGATCTACGGGCRGTGWGTRCA-3′
Second-PCR primers	Forward: 5′AATGATACGGCGACCACCGATCTACA[index1]ACACTCTTTCCCTACACGAC-3′
	Reverse: 5′CAAGCAGAAGACGGCATACGAGAT[index2]GTGACTGGAGTTCAGACGTGT-3′

^a^ Primers used in this work contain Illumina^®^ specific sequences protected by intellectual property (Oligonucleotide sequences © 2007–2013 Illumina, Inc. All rights reserved. Derivative works created by Illumina customers are authorized for use with Illumina instruments and products only. All other uses are strictly prohibited.).

**Table 2:** Human pathogens and antibiotic and zinc resistance genes in the nasopharyngeal flora of pig workers compared to non-exposed controls.

Targeted Pathogenic Agents and Resistance Genes	Controls (*n* = 29) ^a^ %	Pig Farmers (*n* = 25) %
***Staphylococcus aureus* (Firmicutes)**	86.7	100
**MRSA (Firmicutes)**	10	60
***Salmonella* spp. (Proteobacteria)**	80	60
***Clostridium difficile* (Firmicutes)**	0	12
***Mycobacterium avium* (Actinobacteria)**	0	0
***Listeria monocytogenes* (Firmicutes)**	3.33	24
***czrC* (zinc/cadmium)**	26.7	68
***bla*_CTX-M-1_ (cephalosporin)**	0	4
***mcr-1* (colistin)**	0	12

^a^ The results are expressed in percent of subjects in which pathogens or genes were detected.

## Supplementary Materials

The following are available online at https://www.mdpi.com/1660-4601/16/8/1375/s1, Additional file 1 (.xls): Differential abundance of OTUs in the nasopharynx of farmers and non-exposed controls.
